# Genitourinary Tuberculosis: An Atypical Clinical Presentation

**DOI:** 10.1155/2012/727146

**Published:** 2012-12-18

**Authors:** Pei Shan Lim, Ixora Kamisan Atan, Aruku Naidu

**Affiliations:** ^1^Department of Obstetrics & Gynaecology, Pusat Perubatan UKM, Jalan Yaakob Latif, 56000 Cheras, Kuala Lumpur, Malaysia; ^2^Department of Obstetrics & Gynaecology, Hospital Raja Permaisuri Bainun, 30990 Ipoh, Malaysia

## Abstract

Genitourinary tuberculosis is one of the common forms of extrapulmonary tuberculosis. We report a case of atypical genitourinary tuberculosis: massive uterovaginal prolapse with cervical lesion mimicking cervical carcinoma. This particular case highlights the problem of healthcare in most of the developing countries. Lack of patient education, awareness, and access to a healthcare system resulted in a complicated situation. In an endemic area or in an immunocompromised individual, a higher index of suspicion would allow early recognition and treatment institution to minimise its late consequences as well as spreading of the disease. Though anti-TB is the mainstay of treatment, surgical intervention might be needed in selected cases.

## 1. Introduction

Tuberculosis (TB) is caused by *Mycobacterium tuberculosis*. It remains a public health concern especially in developing countries. In Malaysia, the notification of new cases had been steadily increased yearly, and at one point, it was the single important cause of death from notified infectious diseases [1]. Pulmonary infection is generally the main presentation. Extrapulmonary TB is not uncommon especially with the increase in human immunodeficiency virus (HIV) infection. Genitourinary TB is one of the common forms of extrapulmonary tuberculosis, which affects 12% of patients with pulmonary TB [2]. It is more common in women less than 40 years of age and rarely occurs in postmenopausal [3]. We report a case of atypical genitourinary: massive uterovaginal prolapse with cervical lesion mimicking cervical carcinoma.

## 2. Case 

**Figure 1:**
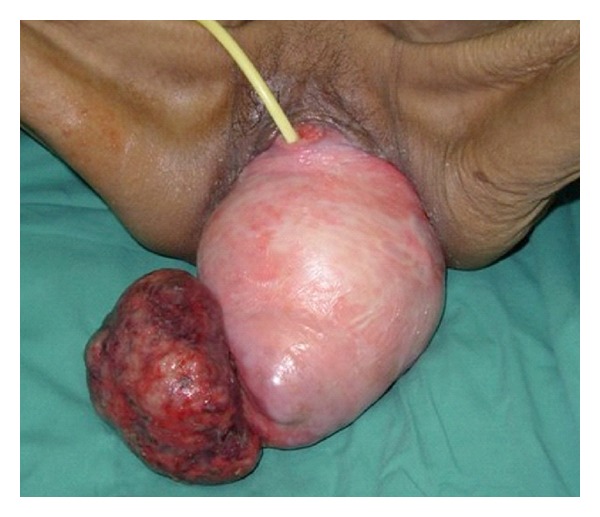
Massive irreducible uterovaginal prolapse with a huge cervical TB.

A 52-year-old para 2, postmenopausal woman, presented with an irreducible uterovaginal prolapse. The prolapse was associated with per vaginal staining, frequency, urgency, nocturia, and voiding difficulty. She had neither past history of tuberculosis nor any contact with TB patients. Clinically she was emaciated and immobile. There was a massive irreducible uterovaginal prolapse with a huge fungating and necrotic mass on the cervix measuring 11 × 9 cm. The growth appeared like a cervical carcinoma (Figure 1). The Chest X-ray was normal. Pelvic ultrasound revealed a solitary bladder stone measuring 3 × 4 cm with no pelvic masses. The biopsy of the growth revealed granulomatous changes and presence of caseating necrosis, which were consistent with TB of the cervix. Her nutritional status was optimised. She underwent vaginal hysterectomy with total colpocleisis and transurethral removal of the bladder calculi. Histology examination of the uterine specimen was consistent with TB of cervix with no uterine involvement. She was subsequently referred to the infectious diseases physician and underwent 9 months of anti-TB treatment.

## 3. Discussion

Although genitourinary TB is one of the common extrapulmonary TB, its actual incidence is difficult to ascertain, as 11% of patients were asymptomatic [4]. Its incidence also varies in different countries. Fallopian tubes' involvement in genital TB was in at least 95–100% of cases and mainly from haematogenous spread [5]. A. M. Sutheland examined 600 women with suspected or proven gynaecological TB in a gynaecological TB clinic in Glasgow, where fallopian tubes' involvement was almost in all cases [6]. Whereas infection of endometrium was encountered in 50–60% of cases, cervical involvement was only 5–15% [5]. Cervical TB is frequently secondary to tuberculous salpingitis and endometritis though direct infection from a partner with tuberculous epididymitis might occur [7]. Gupta et al. performed a review on published cases of cervical TB diagnosed by Pap smear [8]. They found that most cases were in reproductive age group and the presentation often mimics cervical carcinoma due to its ulcerating, fungating, and necrotic appearance. Other possible presentations of cervical TB include papillary, ulcerative, miliary, polypoidal, and increased vascularity and vegetative growth. Though postmenopausal endometrium was thought to be less supportive to tubercle bacilli, our patient was postmenopausal and presented with a huge necrotic cervical mass together with a massive irreducible uterovaginal prolapse.

The diagnosis of genital TB can be established by cytology, histology, and/or bacteriology. Pap smear may offer important provisional diagnosis while histological examination of endometrial biopsy is one of the simplest diagnostic methods. Though culture for TB remains the gold standard of diagnosis, the result was negative in about a third of cases [9]. Other possible aetiologies of granulomas such as lymphogranuloma venereum, sarcoidosis, and schistosomiasis need to be considered when culture is negative. In fact, Usta et al. had reported a case of rheumatoid granuloma of the cervix which gave a similar appearance to TB granuloma [10]. Although the tissue culture was negative in our patient, cervical TB was the most likely cause as Malaysia is an endemic area and she had a positive Mantoux test. 

Management of genital TB includes eradication of the infection and treating its consequences. Combination of antituberculous agents for 9–12 months duration provides more than 95% cure rate [11]. Surgical intervention may be needed, namely, total abdominal hysterectomy and bilateral salpingoophorectomy, if there is persistent or recurrent disease, unhealed fistula, or multi-drug-resistant. Vaginal hysterectomy and total colpocleisis were performed in this patient due to the concurrent massive and irreducible uterovaginal prolapse. Anti-TB treatment was given, as there was no obvious primary source for the cervical tuberculosis. Screening for retroviral infection as well as contact screening was done by the infectious diseases team.

Though genital TB is not common, it remains a contagious disease. The above case represented the consequences of delayed recognition leading to late consequences. It also highlighted the problems of healthcare in most of the developing countries. Lack of patient education, awareness, and access to a healthcare system resulted in a complicated situation. In an endemic area or in an immunocompromised individual, a higher index of suspicion would allow early recognition and treatment institution to minimise its late consequences as well as disease spreading. Anti-TB is the mainstay of treatment. Surgical intervention may be needed in selected cases.
